# Paneth cell adenocarcinoma of the colon: A rare entity

**DOI:** 10.1016/j.ijscr.2019.10.071

**Published:** 2019-11-04

**Authors:** Abdessayed Nihed, Mrabet Soumaya, Baccouche Atika, Ben Jazia Ilhem, Ben Abdelkader Atef, Hmila Fehmi, Mokni Moncef

**Affiliations:** a Department of Pathology, Farhat Hached University Hospital, Sousse, Tunisia; b Department of Gastroenterology, Farhat Hached University Hospital, Sousse, Tunisia; c Department of Surgery, Farhat Hached University Hospital, Sousse, Tunisia; d Research Lab: Transfer in Technology in Anatomic Pathology (LR12SP08), Tunisia

**Keywords:** Paneth, Cell, Colonic, Adenocarcinoma, Pathogenic, Beta-catenin

## Abstract

**Introduction:**

Amongst the morphotypes of colorectal adenocarcinomas, the rich cell type of Paneth constitutes a rare histopathologic variant of adenocarcinoma, which can be observed all along the digestive tract but also in other organs such as the prostate or the breast. About 24 cases were found in the literature, with only 7 cases within the colon and appendix.

**Presentation of case:**

We report the case of a 50-year-old man, without past medical history, complaining of abdominal pain and constipation for 3 months. Biological tests were normal. Radiological investigations and endoscopy revealed a sessile polyp in the right colonic angle measuring 4 cm in greatest diameter. Biopsy concluded to a tubular adenoma with low-grade dysplasia. The patient underwent right hemicolectomy. Microscopically, an invasive adenocarcinoma was identified occupying the colonic mucosal with an invasion of the submucosa. The tumor showed a tubulovillous pattern on the surface and was made mostly of jagged crowded glands in the depth. Some areas exhibit Paneth cell differentiation. No metastatic lymph node was found, and the tumor was staged T1N0. The postoperative course was uneventful. The patient remained free of symptoms at the 6-month follow-up and had no evidence of recurrence.

**Conclusion:**

We reported a Tunisian case of Paneth cell colonic adenocarcinoma. The diagnosis is challenging in biopsies when only well-differentiated areas are sampled. Lysozyme immune-histochemical stain may be helpful when diagnosis difficulty arises. The beta-catenin pathway seems to be activated. More studies are needed for the etiology, pathogenesis, clinical course, prognosis and treatment of Paneth cell carcinoma.

## Highlights

• Colonic Paneth adenocarcinoma is a rare entity with only a few reports in the world literature.
• The pathologist must be aware of the existence of this histological subtype.
• A specific pathogen pathway is incriminated.
• The treatment remains equal to other classic types of colorectal adenocarcinoma.

## 1 Introduction

Paneth cells are unique epithelial cells located at the crypt base of small intestine and proximal colon that play a key role in intestinal homeostasis [1]. Paneth cells are present in chronic non-neoplastic conditions such as inflammatory bowel diseases as well as neoplastic conditions such as adenoma or carcinoma [2]. The prevalence of Paneth cell differentiation in adenomas varies from 0.2 % to 70 % [3]. Its occurrence in carcinomas is rarely reported in gastrointestinal system. Actually, Paneth cell carcinoma raising in a non-inflammatory colonic mucosa, is an exceptional event and to our knowledge, seven cases have been reported in the worldwide literature [4]. The little is known about the relationship between Paneth cell metaplasia and Paneth cell carcinoma nor have any precursor lesions been described [[5], [6]]. Regardless of tumor-genesis pathway, clinical behavior and prognosis remain unclear, due to scarcity of this entity. Through this case report, we will discuss pathologic and clinical features of this particular tumor, emphasizing on pathogenic characteristics. The work has been reported in line with the SCARE 2018 criteria [7].

## 2 Case report

**Fig. 1:**
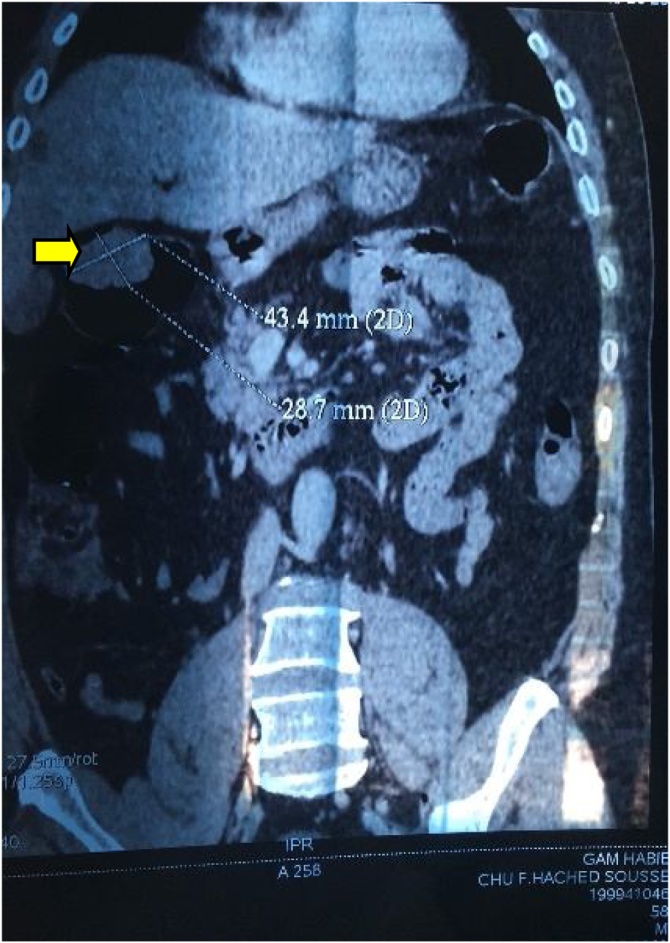
Abdominal CT scan showing a polypoid mass (⇆) in the right colon.

**Fig. 2:**
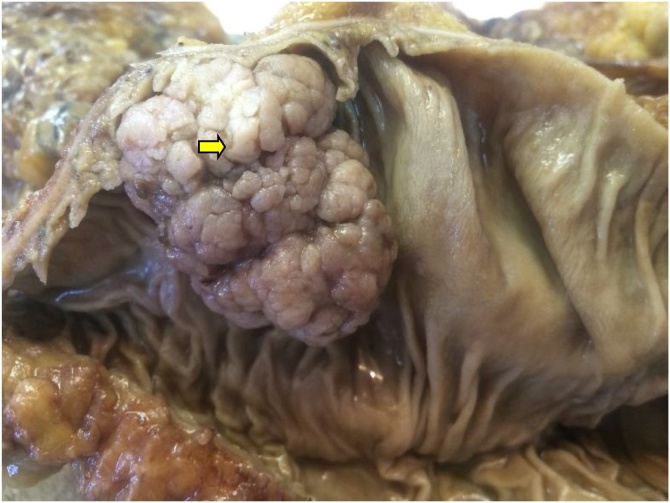
Macroscopic examination of polypoid mass (⇆) with white color and large base of implantation.

**Fig. 3:**
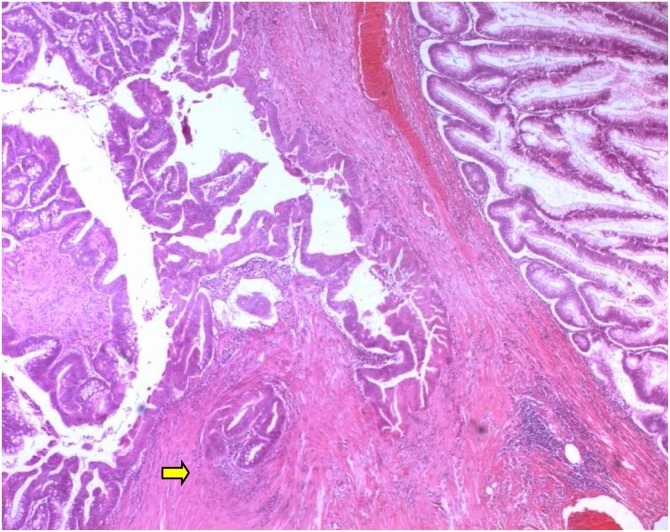
Microscopic finding of the tumor, showing a carcinomatous proliferation well differentiated raising in a high grade adenoma with invasion of submucosa (⇆) (HEx100).

**Fig. 4:**
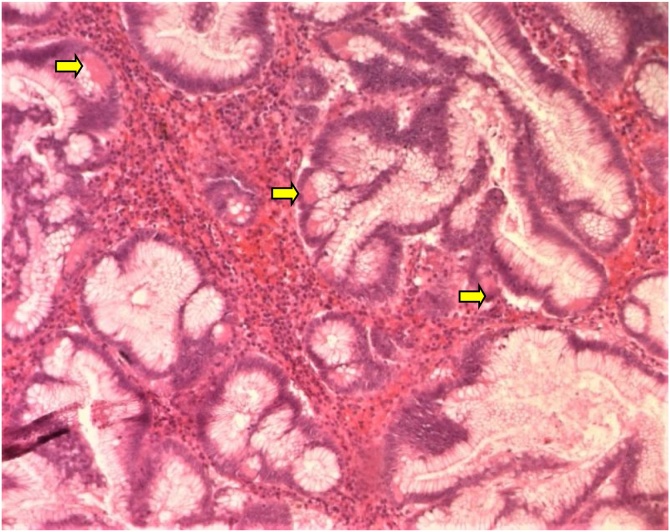
High-power magnification showing crowded tumoral glands of intestinal type with Paneth cell component (HEx200).

**Fig. 5:**
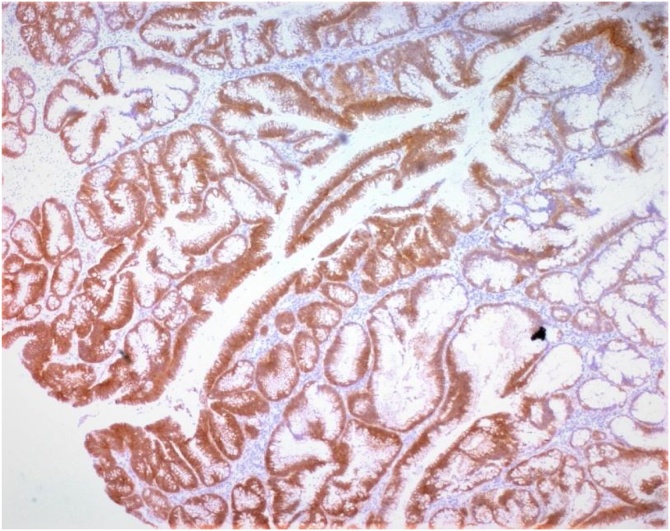
Nuclear b catenin staining at immunochemistry. (IHCx100).

Herein we report the case of a 50-year-old man, without past medical history, presented to our department of gastroenterology with abdominal pain and constipation for 3 months. The abdominal pain was not colicky but progressive and radiated to the epigastric region and relieved spontaneously without analgesics. No similar cases were mentioned in the family, neither any genetic syndrome nor malignancies. No drug history, nor professional exposure were noticed. There was no reported history of vomiting, diarrhea or passage of dark-colored stool and neither weight loss. At physical examination, there were no palpable masses and no collateral findings on the abdominal wall. Biological tests and blood tumor markers were normal. Endoscopy revealed a sessile polyp in the right colonic angle. Biopsy concluded to a tubular adenoma with low-grade dysplasia. The CT scan showed that the mass was measuring 4 cm in greatest diameter, polypoid with a large base (Fig. 1). No other polyp were identified. The patient was transferred to the general surgery department and underwent right hemi colectomy under general anesthesia, by a well-experienced surgeon specialized in operative management of colorectal carcinomas. The surgical procedure turned good without complications, such as hemorrhage, occlusion or peritonitis. On gross examination, the mass was polypoid with a white lobulated surface and large implantation base (Fig. 2). Microscopically, an invasive adenocarcinoma was identified occupying the colonic mucosal with an invasion of the submucosa (Fig. 3). The tumor showed a tubule-villous pattern on the surface and was made mostly of jagged crowded glands in the depth. Some region exhibiting Paneth cell differentiation characterized by an abundant cytoplasm (low nuclear: cytoplasm ratio) containing bright eosinophilic coarse granules and centrally located nuclei (Fig. 4). The transition between the two patterns was gradual with few glands featuring both Paneth cells and mucin secreting cells.There were no specific distribution of Paneth cells, which were observed both on the surface, and in the depth of the tumor. Masson’s trichrome stain highlighted the dense granules within the Paneth cells. At immunochemistry, the tumor show positive nuclear staining with b-catenin antibody (Fig. 5) and a stable microsatellite profile (MSS). Surgical margins were free and no metastatic lymph nodes were found, thus the tumor was staged T1N0. The postoperative course was uneventful. The patient remained free of symptoms at the 6-month follow-up and had no evidence of recurrence. No adjuvant therapy was indicated. However, a close follow-up is planned based on endoscopy and radiologic examinations, to avoid any recurrence and to check new polyps raising. The patient was well informed about his pathology, outcomes and potential risks of recurrence for instance.

## 3 Discussion

Amongst the morphotypes of colorectal adenocarcinomas, the rich cell type of Paneth constitutes a rare histo-pathological variant of adenocarcinoma, which can be observed throughout digestive tract but also in other organs such as the prostate or breast [4].

About 25 cases were found in the literature, distributed evenly between the stomach (8 cases), the small intestine (7 cases) and the colon and appendix (7 cases). For the last three cases, the location is not specified [4]. The mean age of patients is 60 years with extremes of 30 and 89 years [4,[8], [9], [10], [11]]. Clinical symptoms are not specific and even radiologic findings are similar to those observed in classic colonic adenocarcinoma. Histologically, The WHO classification does not define the percentage needed to evoke this diagnosis [12]. The review of the case-rich case histories of Paneth reveals that it is isolated or in 8/25 cases associated with other morphological contingent (colloid and signet ring cells), which is concordant with our case, since we have a predominant well-differentiated component [4].

Paneth cells are normally present in crypts of the glands of the small intestine, appendix, caecum and proximal colon [[8], [9], [10],^12^]. These are typically serous exocrine glandular cells secreting pancreatic-type enzymes (trypsin, phospholipase, etc.) by their zymogen grains. They also possess phagocytic abilities to the intestinal micro-flora by secreting lysozyme and defensins, and playing a key role in bacteriostasis processes [[13], [14]].

Paneth cell metaplasia foci are commonly observed in a wide range of inflammatory, chronic gastrointestinal diseases such as Crohn's disease and ulcerative colitis, but can also be seen in the regenerative mucosa adjoining an adenoma or carcinoma [[1], [2], [3], [4], [5]]. Recent publication demonstrated more frequent K-ras mutation and loss of heterozygosity of microsatellite markers in colonic Paneth cell metaplasia, suggesting Paneth cell metaplasia as a pre-neoplastic lesion [5]. Studies show that the presence of Paneth cells would be associated with an activation of the Wnt /b -catenin pathway suggesting the involvement of this pathway in this differentiation [6]. Even if the presence of this contingent cannot be correlated to the prognosis, given the with a limited number of cases, the presence of the latter must be mentioned in the pathology report in order to recognize it in case of metastases.

## 4 Conclusion

A differentiation of type of Paneth cells can be observed in any benign tumor or inflammatory diseases of the digestive tract. In contrast, Paneth-cell rich adenocarcinoma is a rare histo-pathological subtype that can be found all along the digestive tract, with very rare reported colonic location. Diagnosis is established on the identification of Paneth cells within the tumor; however, there is no cut-off about the Paneth cell component to retain the diagnosis. Through this case report, we emphasize on this particular histologic subtype and its pathogenic pathway.

## Declaration of Competing Interests

The authors have no conflict of interest to declare.

## Funding

This research did not receive any specific grant from funding agencies in the public, commercial, or not-for-profit sectors.

## Ethical approval

Given the nature of the article, a case report, no ethical approval was required.

## Consent

Written informed consent was obtained from the patient for publication of this case and accompanying images. A copy of the written consent is available for review by the Editor-in-Chief of this journal on request.

## Authors’ contributions

Nihed Abdessayed –pathological examination of the specimen and reporting, data collection

Atika Baccouche – Drafting of manuscript, data collection

Ilhem Ben Jazia - Data collection, Editing of the manuscript

Soumaya Mrabet – Data collection, Editing of the manuscript

Fehmi Hmila– Editing of manuscript

Atef Ben Abdelkader – Editing of manuscript

Moncef Mokni –Editing of the manuscript

## Registration of research studies

This does not apply as it is a case report of a patient who has given written consent and has been de-identified. It is therefore not prospective research involving human participant.

## Guarantor

Dr. Nihed Abdessayed.

## Provenance and peer review

Not commissioned, externally peer-reviewed
